# Machine learning-based sales forecasting during crises: Evidence from a Turkish women's clothing retailer

**DOI:** 10.1177/00368504241307719

**Published:** 2025-01-22

**Authors:** Kiymet Tabak Kizgin, Selcuk Alp, Nezir Aydin, Hao Yu

**Affiliations:** 1 Department of Industrial Engineering, 52999 Yildiz Technical University, Istanbul, Turkiye; 2 Department of Statistics, 52999 Yildiz Technical University, Istanbul, Turkiye; 3 College of Science and Engineering, 370593 Hamad Bin Khalifa University, Doha, Qatar; 4 Department of Industrial Engineering, UiT-The Arctic University of Norway, Narvik, Norway

**Keywords:** Crisis period, pandemic, customer behavior, sales forecasting, machine learning

## Abstract

**Background:**

Retail involves directly delivering goods and services to end consumers. Natural disasters and epidemics/pandemics have significant potential to disrupt supply chains, leading to shortages, forecasting errors, price increases, and substantial financial strains on retailers. The COVID-19 pandemic highlighted the need for retail sectors to prepare for crisis impacts on sales forecasts by regularly assessing and adjusting sales volumes, consumer behavior, and forecasting models to adapt to changing conditions.

**Methods:**

This study explores strategies for adapting sales forecasts and retail approaches in response to such crises. By employing different machine learning (ML) methods, we analyze consumer behavior changes and sales impacts across various product categories, including bottom wear, top wear, one piece, accessories, outwear, and shoes during the COVID-19 pandemic.

**Results:**

The gradient boosting and CatBoost algorithms excelled in product groups with significant sales changes during the pandemic. The Multi-Layer Perceptron (MLP) algorithm performed well in low-volume categories like accessories and footwear. Meanwhile, MLP, LightGBM, and XGBoost were effective in medium-volume categories such as outerwear and underwear.

**Conclusion:**

The findings highlight the efficacy of these models in adapting sales forecasts to crisis conditions, offering a practical approach to enhancing retail resilience against future disruptions. This study offers an effective approach for adapting sales forecasting to shifting consumer behaviors during crises.

## Introduction and background

Crisis periods can profoundly affect the retail sector, presenting various business challenges. It is crucial to analyze pre-crisis and crisis period data together to meet customer needs better and make forecasting models sensitive to these changes. The COVID-19 pandemic can be considered the most significant crisis in modern times. 2020 was extremely dynamic for the world economy, with lockdowns, job losses, and supply chain disruptions being among the main issues caused by the pandemic.^2^ During this crisis, COVID-19 also significantly impacted global stock markets.^3^ Experts struggled to obtain reliable economic forecasts, reducing prediction performance.^4^ The retail sector is one of the most adversely affected by the COVID-19 pandemic, facing challenges such as store closures and changing consumer demand.^5^ During COVID-19 restrictions, consumers quickly identified categories like shoes and accessories as “non-essential” and significantly reduced their spending on such items.^6^ This situation has led to issues in inventory management,^7^ pricing, and marketing activities.^8^

A crisis can deeply impact the retail industry, as seen during the COVID-19 pandemic, which caused global disruptions and shifted consumer demand for non-essential items. To navigate such challenges, it is crucial for retailers to understand pre-crisis conditions and employ accurate sales forecasting models. Using machine learning algorithms like Gradient Boosting, CatBoost, and MLP can improve predictions by accounting for rapid changes in consumer behavior. This study combines pre-crisis and crisis data to refine forecasting models, helping retailers better prepare for future crises.

The machine learning (ML) algorithms used in this study were selected to predict consumer behavior and sales trends more accurately during crises. Specifically, algorithms like Gradient Boosting, CatBoost, and Multi-Layer Perceptron (MLP) are known for high performance across different datasets and problem types. There are several reasons for selecting these algorithms: Gradient Boosting and CatBoost perform well on complex and variable datasets. They can capture intricate patterns and accurately predict product categories with high sales volume and variability. Gradient Boosting algorithms can easily adapt to the specific needs of applications by learning according to different loss functions.^9^ MLP algorithm can solve regression and classification problems using artificial neural networks (ANN). This algorithm shows high success in product categories with more straightforward and stable sales patterns. The layered structure of MLP allows for better processing of different features in the dataset.^10^ Mitra et al. (2022) compared five different ML methods (Random Forest (RF), extreme gradient boosting (XGBoost), gradient boosting, adaptive gradient boosting (AdaBoost), and ANN algorithms) and a hybrid model for sales forecasting with weekly sales data of a USA-based retail company.^10^ It is observed that the hybrid model predicts more accurately than the other models according to various performance metrics. Krishna et al. (2018) analyzed forecasting a retail store's sales using different ML techniques.^12^ They presented Regression Analysis and Boosting techniques comparatively and stated that boosting algorithms give better results. Cunha et al. (2023) analyzed the effects of the COVID-19 pandemic on the retail experience of e-commerce customers in the supermarket industry.^13^ Their study aimed to understand how the pandemic has affected customer satisfaction in this sector. Ilieva (2022), in his research on e-commerce customer satisfaction, examined how the COVID-19 pandemic has shaped customer behavior in various retail sectors.^14^ Similarly, studies examining the contribution of machine learning techniques to retail sales forecasting during crisis periods have increased. For example, Sleiman et al. (2022) used machine learning models to forecast sales in the fashion sector in France during the COVID-19 pandemic and analyzed the effects of the crisis in detail.^15^ Likewise, Kim et al. (2023) examined the impacts of the pandemic on different retail categories in South Korea and compared the performance of traditional time series models with machine learning methods.^16^

This study addresses the gap in the literature regarding sales forecasting during crises, as most existing research focuses on normal conditions. The need for this study arises from the inadequacy of traditional models in predicting consumer behavior during unexpected crises like COVID-19. This research demonstrates how machine learning can offer more accurate and adaptable forecasts by integrating pre-crisis and crisis data. In doing so, it fills a critical gap in the literature on crisis management and sales forecasting, helping retail businesses better prepare for future disruptions.

This study combines pre-crisis and crisis (COVID-19) data, offering more accurate results than traditional methods. Data from a women's clothing company with 284 stores and six product categories were used. The primary objective of this study is to analyze changes in consumer behavior in the retail sector during crisis periods (e.g. during the COVID-19 pandemic) and to forecast sales using different machine-learning methods. The study aims to enhance prediction accuracy under crisis conditions by combining pre-pandemic and pandemic data. The research hypotheses include that the accuracy of machine learning models will vary across different product groups during crisis periods and that some algorithms will perform better than others.

The Turkish retail sector was chosen for this study due to its broad and diversified market structure, store types catering to various income groups, and significant shifts in consumer behavior during the COVID-19 pandemic. Turkiye is a major player in the global women's clothing retail sector in terms of production capacity and export potential. As of 2021, it held a 3.7% share in apparel and textile exports, making it the 6th largest exporter in the world.^1^ These characteristics provide an ideal setting for observing the differential impacts of the pandemic on luxury and standard consumption and for analyzing the performance of machine learning algorithms across various product categories.

## Methodology

### Used machine learning algorithms

ML algorithms are widely used in forecasting and problem-solving during various crisis periods. In this study, sales predictions were performed for a company operating in the retail sector with ML algorithms using data from the pandemic period. Gradient Boosting, CatBoost, and LightGBM algorithms from boosting methods, CART algorithm from decision tree methods, MLP from ANN methods, and KNN methods were used to predict sales amounts depending on product groups.

Gradient boosting algorithms are powerful ML techniques that have significantly succeeded in various applications. These algorithms can be easily adapted to the specific needs of applications, e.g. they can be learned according to different loss functions.^11^ CatBoost is a member of boosting ML algorithms and a powerful tool for classification and regression tasks. CatBoost is a suitable algorithm for categorical and heterogeneous data.^17^ Cat-Boost uses weighted voting to increase the weight of data associated with minor errors, improving the predicted results’ accuracy.^18^ LightGBM is a boosting-based algorithm and aims to improve prediction accuracy by combining weak classifiers. Compared to standard boosting tree algorithms, LightGBM uses histogram optimization to segment continuous features, which saves memory and speeds up computation.^19^ The CART algorithm is one of the most successful classification and regression analysis algorithms. It is flexible and robust and does not depend on the distribution type because it is non-parametric.^20^ MLP is a kind of ANN trained using back-propagation algorithms. It can be used to solve both regression and classification problems. In an MLP structure, there are many neurons with different functions. The input layer is the first layer and contains the input variables for processing. Based on the data obtained from the hidden layers, the output layer calculates the output or requested values.^11^ Regression problems are concerned with predicting the outcome of the dependent variable given a set of independent variables. KNN predictions are also based on a voting scheme where the winner is used to label the query.^21^ The methodology of this study involves analyzing pre-crisis and crisis period data to make retail sales forecasts through ML algorithms. The study utilizes data from a retail company operating in Turkiye's women's clothing sector, with 284 stores. The company's data covers six main product types: top wear, one-piece wear, outwear, shoes, accessories, and bottom wear.

### Data management policy

#### Data collection

Sales data were collected daily from January 2017 to December 2022, covering both pre-pandemic (2017-2020) and pandemic (2020-2022) periods. The data includes sales figures from 284 stores in 47 cities across Turkiye, where approximately 67% of the country's population resides. The data includes daily sales figures across six main product categories from 2017 to 2022, covering pre-crisis and crisis periods, including the COVID-19 pandemic. The authenticity and reliability of the data are ensured as it represents real-world sales transactions and store performance over an extended period, providing a solid foundation for the analysis. The company was chosen for its comprehensive sales data, covering various product categories and pre-crisis and crisis periods, offering valuable insights into consumer behavior shifts during the COVID-19 pandemic. While this study primarily focuses on traditional brick-and-mortar retail, the analysis does not include cross-border e-commerce data*.* The exclusion of cross-border e-commerce from the scope of this study is due to the focus on physical store sales data, which provides a more comprehensive and representative analysis of consumer behavior in this context. However, the importance of e-commerce, especially post-pandemic, is acknowledged, and this limitation is noted. In future research, cross-border e-commerce data may be considered for a more holistic analysis. Additionally, three new parameters thought to impact the model were included: GDP per capita, population size of the target audience in the store region, and storage area in the stores as three new independent variables. GDP per capita indicates economic prosperity, directly influencing consumer purchasing power and, consequently, store sales; stores in regions with higher GDP per capita typically achieve greater sales. The population size of the target audience in the store region shapes consumer behavior and store sales by determining the potential customer base; densely populated areas offer higher customer traffic and sales potential. The storage area enhances a store's stocking capacity and product variety, enabling quick responses to consumer demand and supporting sales.

#### Data cleaning and preparation

The data pre-processing steps are as follows:

Step 1: Encoding was conducted for categorical variables such as which brand the store sells and the type of store.

Step 2: GDP per capita in the regions where the stores are located was added to the data as an input variable.

Step 3: The female population aged 18–45 in the target group of the stores was added to the model as an input variable.

Step 4: Warehouse areas of the stores were added to the model as an input variable.

Step 5: 25 product groups were grouped under 6 main product groups.

Pre-processing steps addressed missing data analysis and inconsistencies. Once the data were made suitable for analysis, normalization procedures were conducted. Following this, three ML algorithms known for their high performance across different datasets and problem types were selected: Gradient Boosting, CatBoost, and MLP.

#### Model training and evaluation

The data were divided into training and test sets. The training set was used to train the models, while the test set evaluated their performance. Each algorithm was trained separately for different product categories, with cross-validation to optimize parameters. Model performance was assessed using metrics like R-square, MAE, RMSE, and MSE, and differences between product categories and store types were analyzed. The results highlight the most accurate algorithms, offering valuable insights into changing consumer behavior and helping retailers improve sales forecasts and strategic decisions during crises.

### Performance measurement of models

The difference between classification and regression is the values that the dependent variable can take. In classification, the dependent variable can have only two values, usually coded as 0 and 1, whereas in regression, it has a continuous value. Although regression analysis has been used in many ML studies, there has yet to be a consensus on a standard measure to evaluate the regression results. Many studies use Mean Squared Error (MSE), Root Mean Square Error (RMSE), Mean Absolute Error (MAE), and Mean Absolute Percentage Error (MAPE).^22^ Table 1 shows the metrics used in performance metrics in prediction studies.

**Table 1.** Metrics used in prediction studies.

Resources	Predicted/Forecasted Variable	MSE	RMSE	MAPE	MAE	R^2^	AUC	ROC
Henzel & Sikora, 2020^23^	Promotion efficiency		✓	✓	✓			
Yesilyurt, 2021^24^	Daily river flow		✓		✓	✓		
Park et al., 2023^25^	Wind power outputs		✓		✓			
Shinjae Kim, 2021^26^	Confirmed deaths (COVID-19)		✓		✓			
Szczepanek, 2022^27^	Daily streamflow		✓					
Kang et al., 2019^28^	Social media popularity	✓			✓			
Shi, 2023^29^	Sales			✓				
Ding et al., 2020^30^	Sales		✓					
Gür, 2023^31^	Stock price	✓	✓	✓	✓			
Chen et al., 2024^32^	Sales	✓	✓	✓	✓			
Zhao et al., 2022^33^	Building cooling load		✓					
Ye et al., 2023^34^	Photovoltaic power		✓		✓			
Srivastava et. al, 2019^35^	Solar radiation		✓					
Bao et al., 2023^36^	Forest height		✓			✓		
Zimmerman et al., 2016^37^	Influenza						✓	✓
Abbate et al., 2022^38^	The number of daily orders		✓					
Cordeiro-Costas et al.,2023^39^	Load		✓	✓	✓	✓		
Al-azzawi et al., 2023^40^	Electrical load		✓					
Demir & Citakoglu,2023^41^	Solar radiation		✓		✓	✓		
Haque et al., 2023^42^	COVID-19 Third Wave				✓			
This Study	Pre-post crises sales	✓	✓	✓	✓	✓		

In this study, MAE, MSE, RMSE, R2, and MAPE metrics were used to evaluate the performance of the algorithms.

Mean Squared Error (MSE) is a widely used metric in regression analysis representing the average squared difference between the predicted and actual values. Similarly, Root Mean Squared Error (RMSE) is another common metric that measures the differences between the values predicted by a model (or an estimator) and the values observed).

**R**^2^ is the coefficient of determination that can take values in the range (−∞, 1] according to the mutual relation between the ground truth and the prediction model**.** MAE (Mean Absolute Error) measures the average of the absolute differences between predicted and actual values, while MAPE (Mean Absolute Percentage Error) expresses these differences as a percentage, both serving as metrics to evaluate the accuracy of a predictive model^.^^43^

## Application

### Data collection

This study uses 2017-2022 sales data of a company operating in the retail sector. Monthly sales data for 25 different products were acquired. The retail company sells three brands (I, T, and M). At the same time, the retail company has exclusive (E), high (H), standard (S), and outlet (O) type stores. Raw data are categorized as follows: Store Name (283 stores) as String; Year (2017-2022) as Integer; Month (monthly based) as Integer; Product Type (25 different products) as Categorical; Sales Amount (currency) as Float; Sales Quantity (number of products) as Integer; Indicator (I, T, M) as Categorical; Store Type (E, S, H, O) as Categorical.

### Data analysis

#### Changes to store types

When the data based on store type is analyzed, the average sales in (E)-Exclusive type stores pre-pandemic period was 13409.84. In contrast, the average sales decreased by 23% to 10311.58 during the pandemic. Average sales in (H)-High-type stores decreased by 21% from 9435.38 to 7414.53 during the pandemic. The (O)-Outlet store type experienced a smaller decline, with average store sales falling by 19% from 12357.08 to 9974.471. Average sales in (S)-Standard type stores decreased by 15% to 6750,784 from 7952,456 before the pandemic. (E)-Exclusive type stores experienced the highest decline in average sales, while (S)-Standard type stores experienced the lowest drop. These results indicate that people cut down on their luxury needs more than their standard needs.

#### Changes according to indicator

The change in indicator-based sales of stores in the pre-pandemic period and the pandemic period is given in Table 2.

**Table 2.** Indicator-based average sales (quantity-based) changes.

Indicator	Pandemic	Pre-Pandemic	Change	Direction
ITM	15921,29	12093,94	−0,316470	31% increase
IM	12599,81	14502,64	0,131205	13% decrease
T	6524,112	7735,118	0,156560	15% decrease
O	9974,471	12357,08	0,192813	19% decrease
I	8027,959	10024,98	0,199205	19% decrease
M	18806,12	32046,34	0,413159	41% decrease

When the change in average sales amounts in Table 2 is analyzed, only the stores selling I, T, and M brands simultaneously have seen a 31% increase in average sales amount. The average sales amounts of all other stores decreased. The most significant decrease was in the stores selling the brand, which was selling the most luxurious in type M. Type Stores, with indicator type “O” representing outlet sales. Outlet sales can be one of the three brands. There was a 19% decrease in “O” type sales.

#### Changes according to products

Pre-pandemic and pandemic average sales by product are given in Table 3.

**Table 3.** Changes according to products.

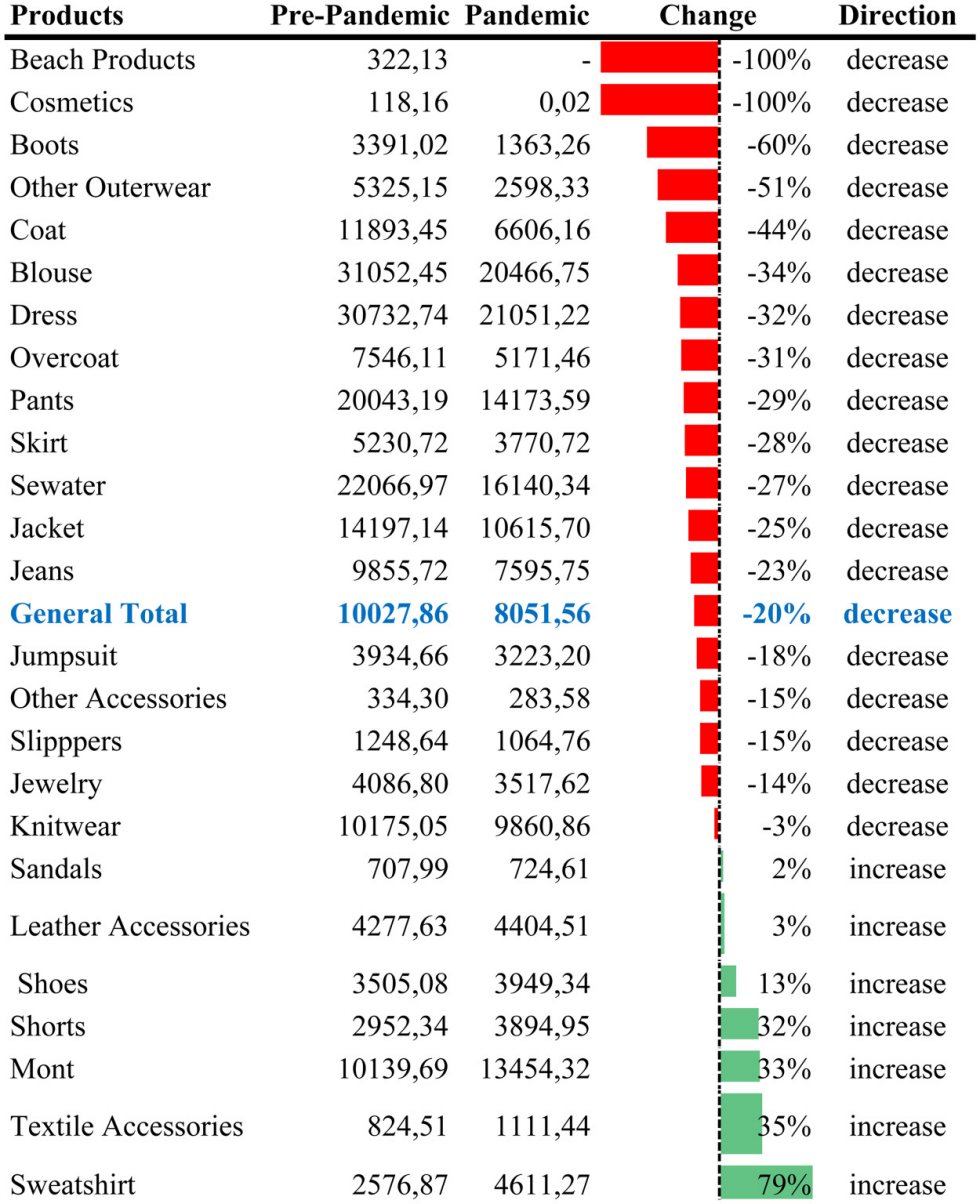

Table 3 shows sales decreased by 20% on average during the pandemic. The decrease in demand for products with above-average decreases shows that there has been a change in the purchasing behavior of customers during the pandemic period. Other outerwear, boots, and cosmetics showed a significant decrease of over 50%. In addition, beach products were not offered to the market by the company, considering that there would be no sales.

While knitwear sales only fell by 3%, much lower than the general decrease, demand remained steady. Sales of sweatshirts and textile accessories (scarves, shawls, hats, etc.) increased by over 35%, reflecting a shift in customer preferences during the pandemic as more people worked from home. Products like overalls, slippers, jewelry, and knitwear saw demand decrease below the average, but the decline was insignificant.

#### Sales prediction by machine learning algorithms

Adebola and Onyekwelu (2019) used ML methods to detect trends in customer behavior.^44^ They developed a model with information such as invoice number, stock code, product information, sales amount, invoice day, unit price, customer number, and country of sale as variables. This study used variables such as product information, product sales amount, and sales amount.

In this part of the study, ML algorithms were used to predict 2023 sales based on 2017-2022 data. Key steps include encoding categorical variables like store type and brand. Additional independent variables, such as store type, monthly product sales, GDP per capita, target group population, and warehouse space, were added to the model. These variables help capture correlations affecting sales. Finally, 25 product groups were categorized into six main groups for analysis: Accessories (Leather, Textile and Other Accessories, Jewelry, Cosmetics, and Beachwear Staff), Bottom wear (Skirts, Jeans, Pants, Shorts), Out wear (Other outwear, Coat, Topcoat), One Piece (Jacket, Dresses, Jumpsuit), Top wear (Blouse, Basics, Sweatshirt, Knitwear), and Shoes (Shoes, Boots, Slipper, Sandals).

Several ML algorithms have been used to model crisis/natural disaster periods. CART,^45^ MLP,^4^ XGBooost,^46^ LightGBM,^47^ Catboost,^48^ KNN,^49^ Gradient Boosting,^50^ and ML algorithms were used for crisis periods such as COVID-19 pandemic, terrorist attacks, and earthquakes. In this study, CART, LIGHTGBM, CatBoost, KNN, Gradient Boost, XGBoost, and MLP algorithms were used as in the above studies. 2023`s sales data were predicted on a product group basis with seven different ML algorithms. 2023`s sales values and the performance measurement metrics calculated accordingly by ML algorithms and error values are presented in Table 4.

**Table 4.** Performance metrics.

**MODELS**					
**CART**	**MAE**	**MSE**	**RMSE**	**R^2^**	**MAPE**
Accessories	0.05	0.005	0.07	0.36	**0**.**26**
Bottom wear	0.12	0.028	0.16	0.36	0.44
Out wear	0.07	0.009	0.098	0.79	0.76
One piece	**0**.**04**	**0**.**003**	**0**.**06**	**0**.**88**	0.48
Top wear	0.06	0.010	0.10	0.62	0.44
Shoes	0.08	0.012	0.1	0.38	0.38

**LIGHTGBM**	**MAE**	**MSE**	**RMSE**	**R^2^**	**MAPE**
Accessories	0.06	0.007	0.08	0.16	0.34
Bottom wear	0.11	0.022	0.15	0.50	0.91
Out wear	0.10	0.02	0.14	0.59	**0**.**11**
One piece	**0**.**04**	**0**.**004**	**0**.**06**	**0**.**84**	0.42
Top wear	0.07	0.014	0.11	0.51	0.87
Shoes	0.08	0.009	0.10	0.52	0.40

**CATBOOST**	**MAE**	**MSE**	**RMSE**	**R^2^**	**MAPE**
Accessories	0.04	0.003	0.05	0.62	**0**.**23**
Bottom wear	0.08	0.017	0.13	0.61	0.51
Out wear	0.064	0.007	0.08	0.84	0.24
One piece	**0**.**02**	**0**.**002**	**0**.**04**	**0**.**92**	0.33
Top wear	0.04	0.003	0.06	0.87	0.48
Shoes	0.06	0.006	0.08	0.65	0.30

**KNN**	**MAE**	**MSE**	**RMSE**	**R^2^**	**MAPE**
Accessories	0.06	0.07	0.08	0.21	0.30
Bottom wear	0.10	0.02	0.16	0.36	0.62
Out wear	0.09	0.015	0.12	0.68	**0**.**25**
One piece	**0**.**04**	**0**.**005**	**0**.**07**	**0**.**81**	0.95
Top wear	0.06	0.008	0.09	0.72	0.75
Shoes	0.09	0.014	0.12	0.18	0.46

**GRADIENT BOOST**	**MAE**	**MSE**	**RMSE**	**R^2^**	**MAPE**
Accessories	0.05	0.005	0.07	0.42	**0**.**25**
Bottom wear	0.10	0.02	0.13	0.60	0.47
Out wear	0.06	0.006	0.006	0.87	0.28
One piece	0.04	0.003	0.05	0.89	0.44
Top wear	**0**.**02**	**0**.**002**	**0**.**04**	**0**.**94**	0.44
Shoes	0.06	0.006	0.08	0.64	0.30

**XGBOOST**	**MAE**	**MSE**	**RMSE**	**R^2^**	**MAPE**
Accessories	0.05	0.005	0.07	0.41	**0**.**28**
Bottom wear	0.10	0.02	0.15	0.50	0.38
Out wear	0.06	0.006	0.07	0.87	0.77
One piece	**0**.**03**	**0**.**002**	**0**.**04**	**0**.**92**	0.41
Top wear	0.04	0.003	0.05	0.88	0.46
Shoes	0.06	0.07	0.08	0.65	0.33

**MLP**	**MAE**	**MSE**	**RMSE**	**R^2^**	**MAPE**
Accessories	0.03	0.003	0.05	0.68	**0**.**20**
Bottom wear	0.07	0.011	0.10	0.74	0.42
Out wear	0.04	0.003	0.055	**0**.**93**	0.5
One piece	0.03	0.004	0.06	0.83	0.37
Top wear	**0**.**02**	**0**.**002**	**0**.**04**	0.92	0.21
Shoes	**0**.**02**	**0**.**002**	**0**.**04**	0.92	**0**.**20**

The values where each model performed the best according to the five performance metrics are highlighted in bold.

When the results in Table 4 are analyzed, the highest R^2^ value was 94% in the Gradient Boosting algorithm in the top wear category product group. On the other hand, the MLP algorithm gave the lowest MAPE value of 0.20 in the shoe product group. CatBoost, Gradient Boosting, XGBoost, and MLP algorithms gave the lowest RMSE value, 0.04.

In the accessories product group, the MLP algorithm gave the best prediction results with a MAPE value of 0.20, and the MLP algorithm had the highest R^2^ value of 0.68. In the Bottomwear product group, the MLP algorithm with an R^2^ value of 0.74, XGBoost with a MAPE value of 0.38, and the MLP algorithm with 0.42 gave the lowest error. In the Outerwear product category, the MLP algorithm had an R2 value of 0.93, and the LightGBM algorithm had a MAPE value of 0.11. In the one-piece product category, the XGBoost algorithm had an R^2^ value of 0.92, and the CatBoost algorithm had a MAPE value of 0.33. In the Topwear product category, significant metric values were obtained by the Gradient Boosting algorithm with an R^2^ value of 0.94 and the CatBoost algorithm with a MAPE value of 0.21. In the Shoe product category, the significant performance metric values belong to the MLP algorithm with an R^2^ value of 0.92 and the MLP algorithm with a MAPE value of 0.20.

**Figure 1.**
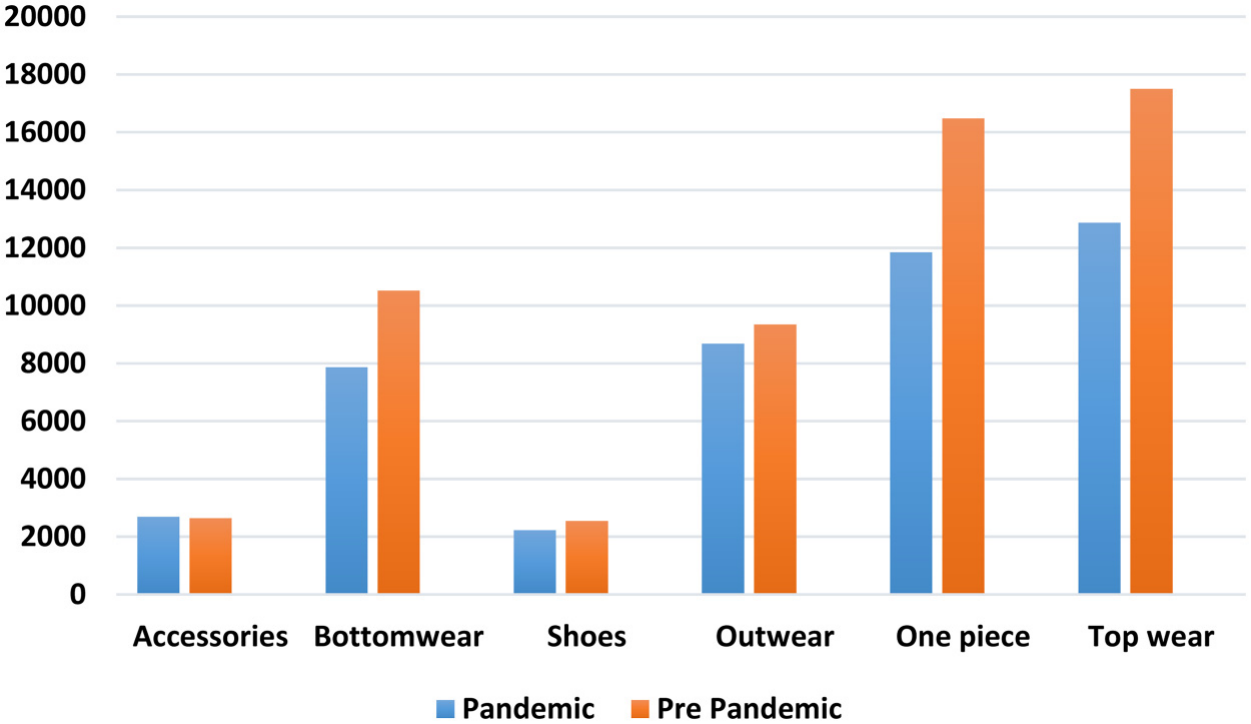
Product-based sales average for pre-pandemic vs pandemic.

Product group-based average sales values for pre-pandemic and pandemic periods were analyzed to evaluate the data better. In Figure 1, product-based average sales amounts are presented.

Figure 1 shows that shoes and accessories had the lowest sales, while topwear and one-pieces had the highest. The largest sales difference between the pre-pandemic and pandemic periods occurred in topwear and one-piece. Gradient Boosting and CatBoost performed best for product groups with the biggest sales changes, while MLP excelled in the accessories and shoes category, where sales remained steady. MLP, LightGBM, and XGBoost performed well for moderate sales changes in outwear and bottomwear.

Comparison of different machine learning algorithms for sales forecasting and their performances are provided in Appendix 1 and 2, Table A1 and Table A2.

Algorithms like Gradient Boosting and XGBoost provide fast and effective results for large datasets, while MLP, a neural network-based method, excels in learning complex data relationships and performs well with large datasets. Although LightGBM delivers fast results on large datasets, it may underperform on smaller ones. CatBoost demonstrates strong results when working with categorical data, whereas the KNN algorithm, despite being simple and interpretable, tends to be slow with large datasets and computationally expensive. The advantages and disadvantages of each algorithm should be considered based on the characteristics of the application and the dataset being used.

Gradient Boosting, MLP, and CatBoost are the algorithms that generally deliver the best performance. Particularly, Gradient Boosting and MLP are notable for their high R2 and low RMSE values in categories with high sales volumes.

Variation by categories: We observe that each algorithm performs better in certain categories. For example, MLP excels in Shoes and Accessories, while Gradient Boosting stands out in the Top Wear and One Piece categories.

Weaker algorithms: KNN and LightGBM generally perform less than other algorithms. Although these algorithms might be more effective with larger datasets, their performance is limited for the data used in this study.

In conclusion, for predicting sales across various product categories in your system, algorithms such as Gradient Boosting, MLP, and CatBoost appear to be the most reasonable choices. Additionally, the high performance shown by algorithms such as Gradient Boosting and CatBoost in certain product groups indicates that retailers could strategically benefit from using these algorithms for inventory management and demand forecasting. This provides an advantage in terms of flexibility and quick response capabilities during crisis periods.

## Discussion and conclusions

In the first year of the COVID-19 pandemic, tens of millions officially contracted COVID-19. This global pandemic has affected the lives of billions of people and has been the subject of academic studies in many different fields. Academic studies have been published on the effects of the pandemic on the retail sector, from stocking products to using personal shopping and distribution services.^51^ Firms that do not consider changing consumer behavior cannot analyze demand trends correctly and fail to respond to customer needs. Companies that cannot make accurate forecasts will encounter difficulties.^52^

This study analyzed product groups affected differently during the pandemic, using sales data from a major women's clothing retailer in Turkiye. Overall sales decreased by 20%, with outerwear, boots, and cosmetics dropping by over 50%. Beach products were not offered for sale. Knitwear saw a smaller decline of 3%, while textile accessories and sweatshirts increased by over 30%. Daily use items like overalls, slippers, and accessories had smaller decreases, reflecting a shift towards practical, everyday clothing over luxury and business attire. Analyzing sales by store type, E-type luxury stores saw a 23% drop, indicating customers avoided luxury purchases during the pandemic. S-type stores offering affordable seasonal products had the lowest sales loss. O-type outlet stores experienced a 19% decrease, while H-type stores, selling more luxurious items, saw a 21% drop. The most significant decline, 41%, occurred in the M brand, which sells luxury fabrics like silk and cashmere. ITM stores, selling all three brands, had a 31% loss due to their indoor locations. Stores selling I and M brands had the least impact, as they catered to middle-income consumers in outdoor shopping centers. Overall, demand shifted towards everyday products rather than luxury or handmade items like silk and cashmere.

Sousa et al. (2023) compared three Gradient Boosting algorithms (CatBoost, LightGBM, and XGBoost) for predicting sales demand for a company in the cosmetics, perfumes, and toiletries market and made predictions for three different periods (one, five, and ten periods, ahead).^53^ XGBoost algorithm performed more consistently in all prediction periods and achieved the lowest error rate. In our study, Gradient Boost, Catboost, and LightGBM algorithms were also selected as boosting algorithms. In general, boosting algorithms performed better in our dataset. MLP algorithm has shown significant success in the accessories and shoes product category with low sales volume change. The gradient boosting and CatBoost algorithms provided better results than others in product groups whose sales volume changes the most. MLP, LightGBM, and XGBoost algorithms performed better in product groups with medium sales volumes, such as outwear and boot wear.

This study offers theoretical contributions to understanding consumer behavior changes and the applicability of sales forecasting models in the retail sector during crises. Unlike previous literature, which often focuses on normal conditions, this research combines pre-crisis and crisis data to show how machine learning methods can generate more accurate forecasts during crises. By analyzing the effects on different product categories, the study provides deeper insights into consumer behavior shifts and contributes significantly to crisis management and sales forecasting literature.

This study's findings indicate that using machine learning algorithms can enhance the accuracy of retail sales forecasting during crisis periods. Retailers can optimize inventory management and improve demand forecasting accuracy by using algorithms such as Gradient Boosting, CatBoost, and MLP, especially during crises. This can aid in making more effective strategic decisions and responding quickly to customer demands. Integrating such models into retail management systems can provide flexibility during fluctuating sales, minimizing revenue losses and increasing customer satisfaction.

## Theoretical contributions

The theoretical contributions of this study stand out through the new perspectives it provides on retail sales forecasting during crisis periods. While the effectiveness of machine learning methods in sales forecasting during the pandemic aligns with previous studies, the novelty of this research lies in combining crisis period data with normal period data to produce more accurate forecasts. Unlike many studies in the literature that focus on forecasting methods under normal conditions, this study offers an integrated approach specifically for crisis periods.

Sleiman et al. utilized sales data from a French fashion retailer for 2019–2020 to model the impact of the pandemic on sales. They focused solely on pandemic data, dividing the pandemic period into three phases: the normal period, the lockdown period, and the recovery period. By implementing their methodology, they conducted short- and mid-term forecasting and compared the error rates of their model with traditional forecasting methods**.**^54^ However, using only pandemic data for forecasting can have a limiting effect in terms of capturing data patterns. Therefore, in this study, a longer period was chosen to include the pandemic period better to capture the impact of the pandemic within the data. Additionally, this study examines how different product groups were affected during crisis periods in detail, addressing product-based impacts often overlooked in the literature. This provides a significant theoretical contribution to crisis management and strategic planning in the retail sector. Kim et al., in their study, aimed to estimate the impact on the retail sector at the sectoral level by analyzing five main retail categories, such as fashion and food and beverages. Our study focused on a single retail sector and aimed to forecast sales within six main product categories, considering the effects of crisis periods**.**^55^ Similar to our study, Krishna et al. examined the impact of the COVID-19 pandemic on a Brazilian fashion retailer's sales using data from July 2018 to August 2022; however, product group-based forecasting was not presented in their study.^56^

## Limitations and future research

This study has some limitations. First, the dataset analyzed belongs to a single women's clothing retailer operating in Turkiye, which may limit the generalizability of the findings to other geographical regions or retail sectors. Second, the study focuses solely on physical store sales data; online sales data were not included in the analysis. Future studies could consider examining the impacts of other factors beyond the pandemic, such as political instability and supply chain disruptions.

## Appendix 1

[Table table5-00368504241307719]

**Table A1. table5-00368504241307719:** Comparison of different machine learning algorithms for sales forecasting.

Algorithm	Description	Advantages	Disadvantages	Speed	Data Size Suitability
Gradient Boosting	Combines many weak learners.	Low error rate, good generalization	Long training time	Medium	Medium
XGBoost	Optimized version of gradient boosting.	Fast, efficient, strong with large datasets	Complex hyperparameter tuning	Fast	Large
LightGBM	Uses histogram-based learning.	Fast with large datasets	May underperform on smaller datasets	Very Fast	Large
CatBoost	Handles categorical features effectively.	Strong with categorical data	Requires extensive hyperparameter tuning	Medium	Medium
MLP	Learn through artificial neural networks.	Capable of learning complex data relationships	Requires large data, long training times	Slow	Large
KNN	Classification based on neighbors.	Simple, interpretable	Computationally expensive, slow on large datasets	Slow	Small/Medium

## Appendix 2

[Table table6-00368504241307719]

**Table A2. table6-00368504241307719:** Algorithm performance comparison.

Algorithm	Accessories (R2, RMSE, MAE)	Bottom Wear (R2, RMSE, MAE)	Out Wear (R2, RMSE, MAE)	One Piece (R2, RMSE, MAE)	Top Wear (R2, RMSE, MAE)	Shoes (R2, RMSE, MAE)
Gradient Boosting	0.42, 0.07, 0.05	0.60, 0.13, 0.10	0.87, 0.06, 0.06	0.89, 0.05, 0.04	0.94, 0.04, 0.02	0.64, 0.08, 0.06
XGBoost	0.41, 0.07, 0.05	0.50, 0.15, 0.10	0.87, 0.07, 0.06	0.92, 0.04, 0.03	0.88, 0.05, 0.04	0.65, 0.08, 0.06
LightGBM	0.16, 0.08, 0.06	0.50, 0.15, 0.11	0.59, 0.14, 0.10	0.84, 0.06, 0.04	0.51, 0.11, 0.07	0.52, 0.10, 0.08
CatBoost	0.62, 0.05, 0.04	0.61, 0.13, 0.08	0.84, 0.08, 0.06	0.92, 0.04, 0.02	0.87, 0.06, 0.04	0.65, 0.08, 0.06
MLP	0.68, 0.05, 0.03	0.74, 0.10, 0.07	0.93, 0.05, 0.04	0.83, 0.06, 0.03	0.92, 0.04, 0.02	0.92, 0.04, 0.02
KNN	0.21, 0.08, 0.06	0.36, 0.16, 0.10	0.68, 0.12, 0.09	0.81, 0.07, 0.04	0.72, 0.09, 0.06	0.18, 0.12, 0.09
